# Macroeconomic-aware forecasting of construction costs in developing countries: Using gated recurrent unit and long short-term memory deep learning framework

**DOI:** 10.1371/journal.pone.0333189

**Published:** 2025-10-09

**Authors:** Majed Alzara, Nadeen Gihad, Heba Abdou, Akram Soltan, AlHussein Hilal, Ahmed Ehab

**Affiliations:** 1 Department of Civil Engineering, College of Engineering, Jouf University, Sakaka, Saudi Arabia; 2 Department of Construction and Building Engineering, College of Engineering and Technology, Arab Academy for Science, Technology and Maritime Transport (AASTMT), Cairo, Egypt; 3 Department of Interior Design, College of Engineering, Jouf University, Sakaka, Saudi Arabia; 4 Department of Civil Engineering, Badr University in Cairo (BUC), Cairo, Egypt; Instituto Nacional de Pesquisas Espaciais, BRAZIL

## Abstract

Cost overruns are common on long-term construction projects. This is mostly because of inaccurate early estimates and unexpected changes in the economy and finances. In Egypt, the costs of materials like steel, cement, bricks, sand, and aggregates make up a large part of the cost of building. These costs are greatly affected by the state of the economy and the financial markets. Even though the Construction Cost Index (CCI) is a widely used economic indicator around the world, Egypt has not yet made its own CCI official. This study creates a predictive model just for Egypt’s construction industry that aims to predict a localized CCI to improve financial planning and lower risk. The framework uses two deep learning models, Long Short-Term Memory (LSTM) and Gated Recurrent Unit (GRU), to make predictions about Egypt’s CCI. The models include a wide range of macroeconomic, monetary, foreign exchange market, commodity/energy market and equity market indicators, as well as technical indicators. In Python, advanced statistical methods like correlation analysis, multicollinearity, and stepwise regression are used to make sure that the best features are chosen. The GRU is better at keeping things in balance because it wins on the calibration (Weighted Absolute Percentage Error (WAPE), Bias (mean error)), the absolute error metrics (Mean Absolute Error, Mean Absolute Percentage Error, Symmetric Mean Absolute Percentage Error, and median error), while LSTM is better at squared-loss/association and turning points (Root Mean Squared Error, Mean Squared Error, Coefficient of determination, Directional Accuracy) because it has a slightly tighter variance fit and sign tracking. There is a permutation feature importance analysis for six features in both the GRU Model and the LSTM Model that shows that oil is the most important thing that affects the construction cost index (CCI). The study shows that deep learning models can accurately predict economic indicators. This gives Egypt’s construction industry a useful, data-driven tool for estimating costs ahead of time. They make a big difference in Egypt’s construction industry and meet the need for localized forecasting models in markets.

## 1 Introduction

Over the last ten years, the Egyptian construction industry has seen substantial ups and downs, mostly due to inflation, frequent currency devaluations, disruptions in the global supply chain, and shifting fuel and material prices. Fuel shortages and EGP depreciation affected material prices to lift in 2014; similar inflationary spikes followed in 2016, 2020, and particularly between 2022 and 2024 following several pound devaluations. The cost of building materials went up significantly because of these changes in the economy, especially for steel, cement, and petrochemicals. Large-scale infrastructure Project like the New Administrative Capital helped bring inflation under control for a while in 2018 and 2019, but continued macroeconomic volatility, including the complete liberalization of the exchange rate in 2024, kept project costs rising. Cost uncertainty is still a problem even with recent indications of currency stabilization and increased foreign reserves, particularly as contractors use price adjustment clauses to protect themselves from exchange-rate volatility.

Even so, Egypt’s construction sector has demonstrated itself to be robust, growing to be a significant contributor to the country’s Gross Domestic Product (GDP) and a top destination for foreign investment. Strategic alliances with Gulf nations, plans for added urbanization, and large-scale housing, transportation, and energy infrastructure projects have improved growth projections. In 2025, the real growth is forecasted to be 6.5% [1]. Nevertheless, cost overruns continue to occur; according to some research, they may reach 55% in residential projects due to inflation, incorrect cost estimates, and rising material prices [2]. To check and forecast changes in material costs, researchers have backed the development of a localized CCI [3].

Commonly usage of the Traditional estimation methods which include (1) unit-rate/detailed quantity take-off and analogous and unit-rate/ARIMA time-series, (2) parametric/regression models, and (3) classical univariate time-series (AutoRegressive Integrated Moving Average (ARIMA)/ Seasonal ARIM (SARIMA)/Holt-Winters). These methods are based on linearity/stationarity assumptions which may not be able to handle the nonlinear, lag, and regime-shifting drivers that are common in construction inputs during macro volatility (Tayefeh Hashemi et al. 2020 [4]; Hyndman & Athanasopoulos, 2021 [5]). Moreover; recent reviews and empirical studies indicate that machine-learning models Artificial Neural Network (ANN)/LSTM and hybrids) frequently achieve great accuracy in predicting CCI (Shamim, 2025 [6]; Aslam et al., 2023 [7]), whereas manual/2D quantity take-off and early analogous/unit-rate techniques struggle amid macro volatility (Liu et al., 2024 [8]). Tayefeh Hashemi et al. (2020) [4] designed the limitations of parametric/regression methodologies, particularly linear-function assumptions, which show suboptimal performance in the presence of nonlinear relationships. Empirical research (Aslam et al., 2023 [7]) shows that nonlinear machine learning (ANN/LSTM) exceeds time-series/regression baselines for CCI/ Highway Construction Cost Index (HCCI), thereby proving the “nonlinear/time-sensitive”. Nonlinear and break-aware models are beneficial within this integrative approach as they more precisely convert alterations in macro drivers into cost outcomes (e.g., AlTalhoni et al., 2024 [9]; Aslam et al., 2023 [7]). Because of this, intelligent forecasting which becomes more required and useful as the economy becomes more unstable, and projects become more complex.

This research focuses on the prediction of the CCI, which is an important indicator for estimating and forecastingof the total project expenses, using complicated machine learning models, particularly Long Short-Term Memory (LSTM) and Gated Recurrent Unit (GRU) neural networks. The complex, nonlinear, and time-dependent nature of construction factors are not covered in the traditional cost estimation techniques, especially in the complex project and in the volatility rise in economic. Therefore, the research employed Python for data preprocessing, processing, and deep learning. It also highlighted the significance of integrating Macroeconomic, Monetary, Commodity/Energy market and Foreign Exchange Market Indicators and technical data to improve model interpretation and accuracy. In a rapidly evolving in construction environment, the suggested deep learning system increases the forecasting accuracy while simultaneously giving decision-makers useful information for strategic planning, forecasting, efficient resource allocation, and strengthen risk management.

The advanced deep learning techniques for cost forecasting in the construction sector are the focus of this study. The following are the primary aims of the study:

I. Forecast accurately Egypt’s CCI considering the country’s unique circumstances.
II. Examine the CCI and a variety of Macroeconomic, Monetary, Commodity/Energy market, Foreign Exchange Market Indicators data indicators to find the correlation that influences changes in construction costs.
III. Prove the usefulness of the suggested deep learning framework in risk assessment, budgeting and forecasting the overall civil work cost with using a sample construction project.

## 2 Literature review

A long-standing problem, accurately estimating construction costs has become even more crucial considering growing economic instability, especially in nations like Egypt. The need for more intricate and adaptable forecasting techniques is highlighted by the volatility of inflation, material price swings, and currency exchange rates. Recent research shows that to enhance prediction performance, there is an increasing focus on combining data and machine learning techniques with Macroeconomic, Monetary, Commodity/Energy market, Foreign Exchange Market Indicators data. In light of prior research and the topics discussed, the literature review is broken down into six main areas: (1)Macroeconomic, Monetary, Commodity/Energy market and Foreign Exchange Market Indicators effecting Cost Dynamics in Egypt; (2) Methods for Estimating and Forecasting Civil Construction Material Prices; (3) The importance of the CCI; (4) statistical techniques made possible by Python; (5) the use of deep learning models, especially GRU and LSTM, for time-series analysis; and (6) feature importance techniques to improve model transparency. The integration of these threads serves as a foundation for building more resilient and contextually aware models that are proper for the changing Egyptian construction industry.

### 2.1 Macroeconomic, monetary, commodity/energy market and foreign exchange market indicators effecting cost dynamics in egypt

There have been many earlier works discussed in these indicators in Table 1 shows a summary of these works with the main kind of each indicator, authors, publication year of each study in Egypt.

**Table 1:** Macroeconomic, Monetary, Foreign Exchange Market, equity market and Commodity/Energy market Indicators in Egypt.

No.	Indicators	Indicators Type	Authors/Year
1	EUR/EGP Exchange Rate	Foreign Exchange Market indicator	Hosny *et al.*2023 [10]
2	USD/EGP Exchange Rate	Foreign Exchange Market indicator	Ramadan 2023 [11]
			Elsaied 2024 [12]
			Hosny *et al.*2023 [10]
			Shiha 2019 [13]
3	GBP/EGP Exchange Rate	Foreign Exchange Market indicator	Hosny *et al.*2023 [10]
4	EGX30 Index	Equity Market Indicator	Ramadan 2023 [11]
			Elsaied 2024 [12]
			ElMalky 2024 [14]
5	Central Bank Interest Rate	Monetary Indicator	Ramadan 2023 [11]
			Hosny *et al*.2023 [10]
6		Consumer Price Index (CPI)	Shiha 2019 [13]
			Ramadan 2023 [5]
			Elsaied 2024 [6]
7	GDP for Construction (GDP)	Macroeconomic Indicator	Ramadan 2023 [11]
			Hosny *et al*.2023 [10]
8	Money Supply (M2)	Monetary Indicator	Shiha 2019 [13]
			Ramadan 2023 [11]
			Elsaied 2024 [12]
			Hosny *et al.*2023 [10]
9	Producer Price Index (PPI)	Macroeconomic Indicator	Shiha 2019 [13]
			Ramadan 2023 [11]
			Elsaied 2024 [12]
			Hosny *et al.*2023 [10]
10	Unemployment Rate	Macroeconomic Indicator	Shiha 2019 [7]
			Ramadan 2023 [5]
			Elsaied 2024 [6]
			Hosny *et al.*2023 [4]
11	Construction Employment Level	Macroeconomic Indicator	Elsaied 2024 [12]
12	Foreign Reserves	Monetary Indicator	Shiha 2019 [13]
			Ramadan 2023 [11]
			Elsaied 2024 [12]
			Hosny et al.2023 [10]
13	Oil Prices	Commodity/Energy market indicator	Ramadan 2023 [11]
			Elsaied 2024 [12]

Table 1 shows the wide range of Macroeconomic, Monetary, Commodity/Energy market and Foreign Exchange Market Indicators that have been used in different research to predict building costs, tender price indices, and other economic factors. These indicators, such as interest rates, exchange rates, inflation rates, and employment levels, show the complicated and multidimensional nature of estimating construction costs. They also show how different methods could be used in different regions and economic situations. This diversity emphasizes the importance of flexible and context-specific forecasting models.

Within the Egyptian context, Recent research has improved data-usage construction cost forecasting. Shiha 2019 [13] showed that (Neural networks can convert macroeconomic indicators into precise material-cost forecasts, while Hosny et al. (2023) [10], who used SPSS for statistical modeling, offered more proof that price fluctuations in building materials are predictable. Extending the scope of method, Elsaied (2024) [12] used a Vector Autoregression (VAR) framework to capture dynamic interdependencies among macroeconomic drivers of Egypt’s CCI, while Ramadan (2023) [11] compared linear and nonlinear approaches—including linear regression, support vector regression, ANNs, and LSTM networks—for forecasting construction indices. Collectively, this literature shows a clear shift toward Artificial Intelligent (AI)-enabled and deep-learning methods for cost forecasting. ElMalky (2024) [14] supported this trend by showing on the EGX30 that a hybrid ARIMA–ANN model can outperform single-paradigm baselines, highlighting the importance of fusing nonlinear representation learning with classical time-series structure. Therefore, these results support the use of complex including hybrid machine-learning architectures and macro-financial indicators to increase the accuracy of CCI forecasting in Egypt.

### 2.2 Methods for estimating and forecasting civil construction material prices

According to Ashworth and Perera 2015 [15], material costs are the reason for the most building expenses and have a significant impact on how accurately forecasting and budgeting the work.

According to Richer 2021 [16], materials form 65–80% of the total construction costs, while labor typically form 20%–35%. According to Abdel-Wahab et al. (2018) [17], material costs can form at least 50% of a project’s overall cost; errors or inconsistencies in material pricing which had a major impact on the overall cost estimation and overrun risk. According to Jayathilaka et al. 2021 [18], labor costs were estimated to account for 20% to 30% of the overall cost overrun, while material costs were estimated to contribute roughly 60% to 70%. Using artificial intelligence-infused cost-revision models, Yazıcıoğlu and Kanoğlu 2020 [19] showed a strong correlation (above 0.9) between foreign exchange rates and the cost of reinforced concrete per square meter in Turkey from 2010 to 2020. Others, like Musarat et al. 2021 [20], showed that inflation results in yearly variations in the cost of construction materials, labor wages, and rental rates for machinery, which causes differences between the project’s original and final budgets.

However, in a study conducted in Hong Kong, Wang et al. 2022 [21] employed Deep Neural Networks and Shapley Additive exPlanations (SHAP) to measure the significant impact of CPI, lending rates, and wage trends on the accuracy of early cost estimation—often more so than project attributes. Because of its strength, accessibility, and adaptability in structural applications, reinforced concrete (RC) continues to be a popular building material worldwide, but especially in the Middle East and Egypt, according to Akal 2023 [22]. A significant amount of the total cost of a construction project is attributed to the cost of RC components, which are mainly steel, cement, sand, and aggregates.

According to Shiha and El-Adawy (2024) [23], material price forecasting generally becomes better when the macroeconomic factors of the significant contributor are considered.

Lastly, from 2020 to 2025, Musarat et al. (2024) [24] created a time-series forecasting model (ARIMA vs. their novel method) to forecast construction rates in Malaysia that considers the. This model included material, labor, and equipment costs. They used the Akaike Information Criterion to show that the model was better than ARIMA.

### 2.3 The Importance of the CCI

One important figure in construction economics is the CCI where Costs are calculated, estimated, and tracked over time. It displays fluctuations in input costs, such as labor, materials, and equipment, and is influenced by several technical, financial market, and macroeconomic indicators. Project planning, contract pricing, cost containment, and financial and risk forecasting all heavily depend on exact CCI, particularly in developing nations where inflation, exchange rate volatility, and market instability are popular.

Other research highlighted the importance of correct CCI forecasting. For instance, research has shown that using machine learning techniques to accurately estimate costs early in a project is important for decision-making. One of the main difficulties in construction management is creating realistic cost models, and many approaches have been considered to increase their accuracy. To forecast the construction costs, Elfahham (2019) [25] compared various machine learning techniques, including neural networks, time series models, and regression approaches. The research focused on how deep learning algorithms can be used to find out the non-linear patterns in economic datasets.

Moreover, Elsaied (2024) [12] created a unique CCI model for Egypt by combining the prices of steel, cement, and bricks with macroeconomic variables like CPI, PPI, money supply, and construction GDP when using machine learning for cost modeling in more ways. The findings showed that the rising prices were affected by monetary and inflationary trends. These nonlinear interactions were more effectively captured by machine learning techniques than by linear models.

Finally, the indirect impact of world oil prices on domestic building costs was investigated by Kamara et al. (2025) [26] where the rising energy prices drive up prices in the construction industry because fuel costs are a part of the transportation and manufacturing of materials. After combining Egyptian inflation and crude price trends, the study concluded that oil prices ought to be considered a strategic forecasting variable.

### 2.4 Statistical methodologies facilitated by python

In the early phases of data science processes, traditional statistical methods remained essential, especially in feature engineering and exploratory data analysis (EDA). In addition to enabling a thorough comprehension of the data, these fundamental methods served as the cornerstone for more complex modeling initiatives. Practitioners can efficiently refine input features and identify data problems early on by combining statistical measures, visualization techniques, and software tools. The quality of later analyses and model development is improved by this synergy between contemporary computational tools and traditional statistics.

Building on the significance of visualization in EDA, Tufte 2001 [27] offered the guidelines for presenting data graphically in an efficient manner. It describes the potent analytical technique of visual inspection. Finding correlations between variables, and understanding data distributions all depend on these methods. Reimann et al. 2005 [28] examined and contrasted several outlier detection techniques, emphasizing the use of graphical tools such as box plots and histograms to find univariate outliers prior to using more intricate multivariate methods.

In support of these visual aids, Mukaka 2012 [29] concluded that linear and monotonic relationships between variables are commonly measured using Pearson correlation and Spearman rank-order correlation. O’Brien 2007 [30] concluded that multicollinearity among predictors, which can otherwise skew model estimates, is commonly detected using the Variance Inflation Factor (VIF). Data preprocessing is thoroughly discussed in Witten et al. 2011 [31]. It covered how to understand data attributes through the practical use of visualization tools like box plots and histograms.

Python’s scientific environment, which comprised pandas (McKinney, 2010) [32], SciPy (Virtanen et al., 2020) [33], and stats models (Seabold and Perktold, 2010) [34], offers a strong foundation for the easy implementation of these methods. In addition, integrating these statistical insights with contemporary machine learning pipelines ensures that models were trained on familiar and preprocessed features, making them more general, practical, and comprehensible. (Kuhn and Johnson, 2013) [35].

### 2.5 Adoption of deep learning models, specifically GRU and LSTM

#### 2.5.1 Gated recurrent unit (GRU) models.

Gated Recurrent Units (GRUs), a kind of Recurrent Neural Network (RNN), were presented by Cho et al. 2014 [36] as a straightforward substitute for Long Short-Term Memory (LSTM) networks. By combining input and forget gates into a single update gate, GRU models solve the vanishing gradient issue and simplify computation. They were particularly helpful in situations with little data or processing power because of their streamlined architecture, which enabled quicker training and more effective memory use.

Even though GRU models have these architectural advantages, the effectiveness of any machine learning model often depends on the scenario in which it is used. Meharie and Shaik (2020) [37], for example, discovered that Random Forest was the most effective machine learning technique for forecasting highway construction cost index (HCCI). This illustrated the significance of selecting the proper model for the dataset and the prediction task. Additionally, it meant that other machine learning models were also being actively studied and investigated, and that GRUs were only one of many potential models.

Nevertheless, in several series forecasting applications, GRUs have outperformed alternative techniques. When predicting highway speeds, Jeong et al. 2021 [38] employed a GRU model, which they found to be faster to compute and more exact than both ARIMA and LSTM models. By using a GRU-based approach for stock prediction that combined information from industries and an auxiliary module for dataset reconstruction, Chen et al. 2023 [39] provided support for these findings. Their model significantly improved exact prediction across many industries by improving both feature representation and generalization.

More evidence that GRUs are practical comes from Deng et al. 2023 [40], who looked at Beijing’s road performance. According to their findings, GRU models needed significantly less training time than LSTM models while keeping an accuracy level that was comparable. The challenge of predicting the costs of building materials, which were highly unstable and did not follow a straight line, was finally discussed by Guo et al. 2025 [41]. Their suggested hybrid framework—which included GRU networks—performed better than benchmark and standard models, enhancing GRU’s standing as a flexible and multi-domain prediction tool.

#### 2.5.2 Long short-term memory (LSTM) models.

A recurrent neural network (RNN) of the Long Short-Term Memory (LSTM) type was created by Hochreiter and Schmidhuber in 1997 [42]. Because they have internal memory gates that support the vanishing gradient problem, they excel at processing sequential and time-series data. In the construction industry, where material costs, macroeconomic considerations, and time constraints are crucial, LSTM has emerged as a popular architecture for cost prediction.

According to preliminary research, LSTM could improve the accuracy of construction project predictions. For example, Dong et al. 2020 [43] used historical pricing data and LSTM networks to forecast construction cost indices in China. When they compared the LSTM, SVM, and basic RNN models, they discovered that the LSTM model performed significantly better in terms of Root Mean Square Error (RMSE) and Mean Absolute Percentage Error (MAPE).

Building on these initial achievements, added research has changed LSTM models to function in various locales and circumstances. For example, Ramadan 2023 [11] forecasted Egypt’s Construction Price Index (CPI) using both LSTM and conventional models. The money supply, GDP, inflation, interest rates, exchange rates, and the stock prices of construction companies were all examined in this study. The multidimensional model that resulted linked the price of materials (such as scaffolding, steel, and cement) to financial and economic considerations and provided a helpful standard for CPI modeling.

But LSTM is not limited to forecasting the economy; it can also forecast the behavior of structures. A novel framework using LSTM layers in conjunction with genetic algorithms, parametric modeling, and finite element analysis (FEA) was presented by Ghaffari et al. in 2024 [43]. This model effectively stood for the time-varying displacements of high-rise buildings with a low error rate (MSE = 0.0033) and a highly exact prediction (R2 = 0.9939). This showed the potential value of LSTM for structural health monitoring (SHM) in complex seismic or load scenarios. With predictions that were only 0.93% off in the vertical direction and less than 7.5% off in the lateral direction, the model showed its high accuracy and versatility. The present study shows the potential utility of LSTM-based models for structural health monitoring (SHM), particularly for forecasting real-time displacements in high-rise structures subjected to seismic activity or complex loading.

By combining several techniques, recent advancements have increased the power of LSTM [44]. Wang and Qiao 2024 [45] developed a model that makes use of an enhanced form of particle swarm optimization (PSO), an attention mechanism, and Bidirectional LSTM (BiLSTM). This hybrid model dramatically reduced the Mean Absolute Percentage Error (MAPE) in forecasting complex projects when paired with optimization and attention-based innovations. This showed that LSTM was adaptable and performed better when combined with these novel concepts. Forecasts were significantly more accurate using this method, particularly for projects affected by shifting circumstances and economic conditions.

Lastly, comparative evaluations proved LSTM’s advantages, particularly for large-scale applications. Nineteen Earned Value Management (EVM) models, including ANN, SVM, RF, and LSTM, were compared by Yalçın et al. [46] in 2024. Long-term, large-scale construction projects showed LSTM’s stability and scalability in real-world scenarios and applications, while ANN performed marginally better on smaller datasets.

### 2.6 Importance of permutation features in boosting model transparency

Scikit-learn, 2025 [47] showed the Permutation Feature Importance (PFI), a popular technique for figuring out the relative importance of various input variables in the prediction model without the need for a particular model. By methodically altering one feature’s values at a time and calculating the resulting decline in model performance, PFI calculated the extent to which each variable influences a model’s accuracy. In domains like construction cost forecasting, where understanding the impact of features on the model was crucial for making informed decisions and having faith in its results, this method made it simpler to understand and see through complex machine learning models.

### 2.7 Combined approaches

Combining machine learning and statistical preprocessing is emphasized in recent studies. Prior to training ML models, hybrid material-price workflows also often use multicollinearity checks, correlation screening with lags, and parsimonious variable selection (e.g., stepwise regression); in construction-materials studies, both Pearson and Spearman correlations are used in conjunction with stepwise selection (AKL,2023) [22]. For instance, in Egypt: Shiha (2019) [13] applies this logic from beginning to end in Egypt: (i) Pearson and Spearman tests with time lags are used to identify the leading macro-indicators for steel/cement prices; (ii) multicollinearity among predictors is removed; (iii) stepwise regression is used to retain variables that maximize adjusted R2 while minimizing AIC; and (iv) three ANN implementations (Excel+GA, Neural Tools, and Python) are trained. The Python ANN performed the best in tracking month-to-month steel price movements (MAPE ≈ 10.1% test), while the Excel ANN achieved MAPE ≈ 8.74% for cement. These results show a statistical-ML hybrid clearly outperform linear baselines. Decomposition-plus-DL hybrids, in addition to feature-engineering and selection, further improve accuracy for construction indices. For instance, a VMD–LSTM/GRU model for the Texas HCCI breaks down the signal into IMFs and distributes LSTM to smoother components and GRU to volatile ones. This shows how statistical structure can improve deep sequence learners on nonstationary cost data (Wang et al., 2025) [48].

## 3 Methodology

**Fig 1:**
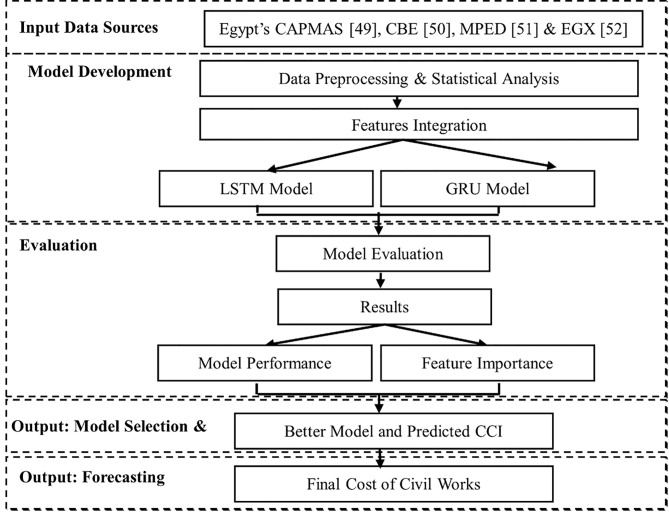
Research Methodology.

The method employed in this research is carefully thought out to yield precise construction cost index forecasts. It begins with the data’s sources. After this data has been cleaned up and statistically examined, GRU and LSTM models are developed using the permutation feature importance, which improves and qualifies model inputs. These models are compared to decide their predictive power and identify the salient characteristics that affect performance. The best model is then chosen to predict the ultimate cost of civil work. (Fig 1) provides a summary of this methodological framework.

### 3.1 Data & CCI formulation

This study uses extensive data collected from Egypt’s Central Agency for Public Mobilization and Statistics (CAPMAS) [49], including the cost of building materials like steel, cement, bricks, sand, and aggregates, as well as important macroeconomic data like the CPI, PPI, unemployment rates, and construction employment levels, as well as commodity/energy market data like oil prices. And Monetary and foreign exchange market indicators from Central bank of Egypt (CBE) [50], such as the EUR/EGP, USD/EGP, and GBP/EGP exchange rates); GDP for construction from Ministry of Planning and Economic Development (MPED) [51]. as well as technical indicators, such as the Relative Strength Index (RSI), various Exponential Moving Averages (EMAD, EMAM, and EMAS), and Bollinger Bands, are gathered. Egyptian Exchange Rate is collected from The Egyptian Exchange (EGX) [52]. The data was subjected to extensive preprocessing to resolve temporal inconsistencies, standardize formats, and get it ready for efficient empirical modeling to guarantee analytical accuracy.

A weighted combination of important construction material price ratios is used to calculate the CCI, which highlights variations from the base-year values of 2014. Equations (1), (2), and (3) provide a mathematical expression for this relationship in period t.


$$CCI_{t}=\sum_{i}w_{i}\;\text{x}\;\left(\frac{p_{it}}{p_{i\text{0}}}\right)$$ (1)

Where:

$CCI_{t}$: Construction Cost Index at time t (dimensionless; often scaled so CCI₀ = 1).

t: Time period

0: Base year.

i: Material indices running over the set of included materials (e.g., steel, cement, bricks, sand, aggregate1, aggregate2)

∑ i: Sum over all Materials i included in the basket

$w_{i}$: Fixed base-year weight of Material i (expenditure share); satisfies $\Sigma_{i}w_{i}=\;\text{1}$

$p_{it}$: Price of Material i at time t

$p_{i\text{0}}$: Price of Material i in the base period.

$\frac{p_{it}}{p_{i\text{0}}}$: Price relative for Material i (dimensionless).


$$\mathrm{\backslash footnotesize}\begin{array}{cc} CCI_{t}=\;\; & w_{steel}\;\text{x}\;\left(\;\frac{p_{\text{steel},\text{t}}}{p_{\text{steel},\text{0}}}\right)+\;w_{cement}\;\text{x}\;\left(\frac{p_{\text{cement},\text{t}}}{p_{\text{cement},\text{0}}}\right)\;+w_{bricks}\;\text{x}\;\left(\frac{p_{\text{bricks},\text{t}}}{p_{\text{bricks},\text{0}}}\right)+w_{sand}\text{x}\left(\;\;\frac{p_{\text{sand},\text{t}}}{p_{\text{sand},\text{0}}}\right)\;\;\;\\ & +w_{\text{aggregate1}}\text{x}\left(\;\;\;\frac{p_{\text{aggregate1},\text{t}}}{p_{\text{aggregate1},\text{0}}}\right)\;\;+w_{\text{aggregate2}}\;\text{x}\;\left(\;\;\;\frac{p_{\text{aggregate2},\text{t}}}{p_{\text{aggregate2},\text{0}\;}}\right) \end{array}$$ (2)

Where:

$CCI_{t}$: Construction Cost Index at time t (dimensionless; often scaled so CCI₀ = 1).

t: Time period

0: Base year.

$w_{steel}$, $w_{cement}$, $w_{bricks}$, $w_{sand}$, $w_{\text{aggregate1}}$, $w_{\text{aggregate2}}$: Fixed base-year weights (expenditure shares) for each material; satisfy


$$w_{steel\;}+\;w_{cement}+w_{bricks}+w_{sand}+w_{\text{aggregate1}}+w_{\text{aggregate2}}=\text{1}.$$

$p_{\text{steel},\text{t}}$, $p_{\text{cement},\text{t}}$, $p_{\text{bricks},\text{t}}$, $p_{\text{sand},\text{t}}$, $p_{\text{aggregate1},\text{t}}$, $p_{\text{aggregate2},\text{t}}$: Unit prices at time t for the respective materials.

$p_{\text{steel},\text{0}}$, $p_{\text{cement},\text{0}}$, $p_{\text{bricks},\text{0}}$, $p_{\text{sand},\text{0}}$, $p_{\text{aggregate1},\text{0}}$, $p_{\text{aggregate2},\text{0}}$: Unit prices in the base year for the respective materials.

The weights in Equation (3) are base-year value shares (Laspeyres-type construction). For each material iii, we first compute its base-year cost (price × quantity) and then divide by the sum across all materials. Concretely, using 2014 as the base year:

For each material, the base-year quantity $q_{i\text{0}}$ is the *Total Industrial Quantity in* 2014.


$$w_{i}=\frac{\left(p_{i\text{0}\;}\text{x}\;q_{i\text{0}}\right)}{\Sigma_{j}\left(p_{j\text{0}\;}\text{x}\;q_{j\text{0}}\right)}\;\;\;\;\;\;with\;\;\;\;\Sigma_{i}w_{i}=\;\text{1}\;\;\;\;$$ (3)

Where:

$w_{i}$: Weight of material i in the index

$p_{i\text{0}}$: Unit price of material i in the base year (the subscript 0 denotes base year, e.g., 2014).

$q_{i\text{0}}$: Quantity of Material i in the base year.

$\;\Sigma_{j}\left(p_{j\text{0}}\;x\;q_{j\text{0}}\right)$: Sum of base-year expenditures multiplying production over all Materials j (denominator).

${\;\Sigma }_{i}\;w_{i}$: 1 (Weights across all items sum to 1)

 i,j: Material indices running over the set of included materials (e.g., steel, cement, bricks, sand, aggregate1, aggregate2).

This formula reflects the proportional impact of material price fluctuations on the overall CCI, showing a foundation for forecasting construction cost trends over the time as shown in the final equation (4):


$$\mathrm{\backslash footnotesize}\begin{array}{cc} CCI=\;\; & \text{0}.\text{5}\text{3}\text{1}\text{0}\text{5}\text{6}\times \left(\frac{p_{\text{steel},\text{t}}}{\text{5},\text{200}.\text{08}}\right)+\text{0}.\text{4}\text{6}\text{6}\text{8}\text{6}\text{2}\times \left(\frac{p_{\text{cement},\text{t}}}{\text{3}\text{7}.\text{4}\text{9}\;}\right)+\text{0}.\text{0}\text{0}\text{0}\text{3}\text{3}\text{6}\times \left(\frac{p_{\text{bricks},\text{t}}}{\text{346}.\text{64}}\right)+\text{0}.\text{0}\text{0}\text{1}\text{4}\text{2}\text{7}\times \left(\frac{p_{\text{sand},\text{t}}}{\text{36}.\text{94}}\right)\\ & +\text{0}.\text{0}\text{0}\text{0}\text{1}\text{6}\text{2}\times \;\;\left(\frac{p_{\text{aggregate1},\text{t}}}{\text{82}.\text{96}}\right)+\text{0}.\text{0}\text{0}\text{0}\text{1}\text{5}\text{7}\times \left(\frac{p_{\text{aggregate2},\text{t}}}{\text{8}\text{0}.\text{3}\text{3}\;}\right) \end{array}$$ (4)

### 3.2 Data interpolation and smoothing

The dataset augmentation technique, which is a part of data interpolation, enhances the consistency and quality of time series data for material pricing, monetary, macroeconomic, foreign exchange, and equity market indicators. By significantly reducing the random fluctuations while keeping the underlying patterns, the data smoothing technique improved the accuracy, clarity, and understandability of the research.

Our preprocessing pipeline is designed to sequence all series on a common calendar, impute only within-sample gaps in a time-respecting manner, attenuate high-frequency noise while maintaining turning points, and document diagnostics to stop information from leaking into model evaluation.

#### (1) Harmonization of the calendar and frequencies.

In order to reindex them to a single target frequency (daily when computing technical indicators; otherwise, monthly), we convert all indicators to a “Date time Index.” The complete span [min (Date), max (Date)] is used for reindexing. For principled time-series interpolation, reindexing establishes explicit gaps at missing timestamps (Hyndman and Athanasopoulos 2021 [5]; pandas development team, n.d.-a [53]).

#### (2) Interpolation (no extrapolation, only within-sample).

Time-weighted linear interpolation (method = “time”) fills in internal gaps by avoiding the equal-step assumption of simple linear methods and respecting unequal spacing in the “Date time Index” (pandas development team, n.d.-b [54]). Piecewise Cubic Hermite Interpolating Polynomial (PCHIP) is used as a documented alternative for variables that need to stay shaped or monotonic-preserving (e.g., some stock measures) (SciPy Developers n.d.-a [55]). There is never any end-point extrapolation or interpolation beyond the observed support.

#### (3) Smoothing, which attenuates noise while keeping peak quality.

We use a local least-squares polynomial smoother, or “Savitzky–Golay (SG)” filter, once a complete within-sample series has been obtained. This is because, in contrast to moving-average smoothers, it reduces high-frequency noise while keeping the height and timing of peaks and inflection points (Savitzky and Golay, 1964 [56]). To ensure stable boundary handling, SciPy’s “savgol_filter” is used with “window_length” greater than polyorder and “mode=”interp”; parameters are adjusted in a narrow grid and examined for over-smoothing (SciPy Developers n.d.-b [57]; Virtanen et al., 2020 [33]).

#### (4) Reproducibility, safety, and diagnostics.

The output consists of turning-point alignment checks, overlay plots of the original and smoothed series, and a basic fidelity metric to identify over-smoothing. To guarantee precise reproducibility, the library versions are documented, and any optional noise augmentation is eliminated from the pipeline. To guarantee total reproducibility, all parameters (frequency, interpolation technique, SG window, and order) are version-controlled using the Python code. McKinney, 2010 [32]; Virtanen et al., 2020 [33].

### 3.3 Statistical analysis with python

To examine the relationships between Egypt’s CCI and indicators of the macroeconomic, monetary, equity, foreign exchange, and commodity/energy markets, a comprehensive statistical analytical framework was developed in Python. To guarantee numerical consistency, the daily time-series dataset was undergoing a thorough preprocessing process. Lagged variables were introduced at 0, 1, 3, and 6 months. Hyndman and Athanasopoulos 2021 [5] acknowledged techniques in time-series analysis forecasting and control, such as accounting for time-based dependencies, which increase the analysis’s reliability. Virtanen et al., 2020 [33]) quantified the associations using the Spearman’s rank correlation to identify monotonic but nonlinear trends and the Pearson correlation coefficient to identify linear relationships. These calculations were performed using the SciPy package, and Pandas was used to manage and format the data (McKinney, 2010 [32]). To make visualizations easier to understand, Hunter (2007) [58] used Matplotlib to create scatter plots.

In order to illustrate the relationships between the variables, a correlation matrix was constructed and displayed as a heatmap. A grid with colors that indicate the strength and direction of correlations is called a correlation heatmap. Strong correlations were shown by dark colors, negative correlations by cool colors, and positive correlations by warm colours [59].

In order to handle multicollinearity, O’Brien 2007 [30] suggested a method for calculating the Variance Inflation Factor (VIF) values and supported it with pairwise Pearson correlation analyses; variables with |r| > 0.8 indicate strong collinearity.

The statistically significant factors influencing the CCI were then shown using a forward stepwise regression technique. Until no more contributors satisfied the requirements or the maximum number of steps (20) was reached, variables were added repeatedly based on p-value thresholds (p < 0.05). A parsimonious and predictive model was produced by Burnham and Anderson (2002 [60]), who used R2, adjusted R2, Akaike Information Criterion (AIC), and Bayesian Information Criterion (BIC) to analyze model performance. Ordinary Least Squares (OLS), which was used to estimate the final model, offered a strong statistical basis for interpretation.

### 3.4 Modelling

This study creates forecasting models for the CCI using two sophisticated varieties of Recurrent Neural Networks (RNNs): Gated Recurrent Unit (GRU) and Long Short-Term Memory (LSTM). The workflow is configured using a structured Python script. It entails configuring the environment, preparing the data, creating input sequences, constructing and training models, forecasting future values, assessing performance, and displaying the results visually.

#### 3.4.1 Configuring the environment.

Python, which has many libraries for data processing, analysis, and deep learning, is used to create both the GRU and LSTM models. While pandas_ta is used to identify technical indicators, pandas and NumPy are used to work with data and numerical operations. While Scikit-learn tools are used to handle preparation tasks like data splitting, encoding, and normalization, Matplotlib and Seaborn are used to aid in visualization. Deep learning architectures are constructed using Keras and comprise Dense, Input, Activation, and GRU or LSTM layers. Keras optimizers are used to improve their performance. Warning: To ensure a cleaner runtime, suppression was used. In particular, the Keras Early Stopping callback stops training when validation performance stops improving, preventing overfitting for the LSTM model.

#### 3.4.2 Preparing and loading data.

The dataset is first imported into a Panda DataFrame from an Excel file for both the GRU and LSTM models. The ‘Date’ column is then transformed into a datetime format for time-series analysis. To improve the dataset, technical indicators like RSI and EMAs (25, 100, and 150) are calculated. The original “CCI” column is eliminated and replaced with a new target variable called “Target Next Price,” which stands for the CCI value for the following day. By removing missing values and restarting the index, the data is cleaned. All features are normalized to a range of 0–1, and categorical variables are numerically encoded. To be used in the forecasting models, the final processed dataset is subsequently saved to a fresh Excel file.

Furthermore, A custom create_sequences function is created to generate input-output pairs using sliding windows over the multivariate time series data in order to support temporal learning in both the GRU and LSTM models. Thirty-five earlier steps of contributor variables were included in each input sequence, and the target value that followed the sequence was the corresponding output. The input features do not include the target variable. The data is split into 90% training and 10% testing sets using a non-random, time-based split, which disables shuffling to keep the chronological order.

#### 3.4.3 Establishment and training.

Using GRU and LSTM architectures, a deep learning framework is put into practice to model temporal dependencies in time series data spanning January 2008 through September 2024. Three GRU layers (750, 550, and 350 units) are used in each model’s stacked structure. The first two layers returned sequences, while the last one generated a vector. A dense layer with ReLU activation comes next. The Adam optimizer is used to build models, with an MSE loss and a learning rate of 0.0005. It had an early stop to minimize overfitting and restore the correct weights after being trained for 50 epochs with a batch size of 25 and validated at 10%. Batch-level shuffling and the Keras fit() method are used to train both models between January 2008 and December 2022. Although Both models are tested between January 2023 and September 2024.Moreover, the performance of the prediction of the GRU and LSTM models is examined, analyzed, and compared using a thorough evaluation technique. To obtain quantitative data and insights, each model is tested separately using standard error metrics. The CCI forecasts are then visually assessed to guarantee their accuracy and interpretability.

### 3.5 Denormalization

This study applies a reproducible post-processing pipeline that uses a back-transformation learned on the overlap window between normalized model outputs and raw CCI to return LSTM Model and GRU Model outputs to the native CCI scale. The mapping y=ax+b is specifically estimated using ordinary least squares (OLS) from normalized Actual values (x) to observed CCI (y); the slope a and intercept b that result serve as the empirical inverse of common normalizations (e.g., a≈σa, b≈μ for z−score; a≈max−min,b≈min for min−max).This transformation is applied to both series to obtain denormalized values, from which residuals (Predicted − Actual) are calculated in CCI units, assuming that Actual and Predicted share a scaler. Overlap $R^{\text{2}}$ is used to verify the calibration quality. For complete reproducibility, the pipeline uses a single main () function to embed three diagnostics figures (time series, residuals over time, and Predicted-vs-Actual with the y=x line) and export an auditable Excel package (denormalized data, diagnostics, sample alignment).

### 3.6 Evaluation metrics

Using complementary error metrics that capture various aspects of accuracy and reliability on the data, a thorough evaluation of the CCI forecasting models was carried out. Typical absolute deviations are summarized by Mean Absolute Error (MAE) and Median Absolute Error (MedAE), with MedAE which is being resistant to outliers. Both Mean Squared Error (MSE) and Root Mean Squared Error (RMSE) highlight larger errors and, in the case of Root Mean Squared Error (RMSE), keep the target’s original units. The percentage of variance that the model explains is measured by the coefficient of determination (R²). Comparability between series is eased by scale-free percentage measures, such as (Mean Absolute Percentage Error (MAPE)and Symmetric Mean Absolute Percentage Error (sMAPE), the latter of which is symmetric and bounded. The model’s ability to accurately forecast the direction of change from one period to the next is measured by its Directional Accuracy (DA). Bias (mean error) denotes systematic under- or over-prediction, while Weighted Absolute Percentage Error (WAPE) stands for the total absolute error in relation to the series’ aggregate level. Equations (5) through (14) which provide formal definitions.


$$MAE=\frac{\text{1}}{n}\sum_{i=\text{1}}^{n}\left|y_{i}-\hat{y_{i}}\right|$$ (5)

$$MedAE=median(\left|y_{i}-\hat{y_{i}}\right|)$$ (6)

$$MSE=\frac{\text{1}}{n}\sum_{i=\text{1}}^{n}{\left(y_{i}-\hat{y_{i}}\right)}^{\text{2}}$$ (7)

$$RMSE=\sqrt{\frac{\text{1}}{n}\sum_{i=\text{1}}^{n}{\left(y_{i}-\hat{y_{i}}\right)}^{\text{2}}}$$ (8)

$${\text{R}}^{\text{2}}=\text{1}-\frac{\sum_{i=\text{1}}^{n}{\left(y_{i}-\hat{y_{i}}\right)}^{\text{2}}}{\sum_{i=\text{1}}^{n}{\left(y_{i}-\overset{―}{y}\right)}^{\text{2}}}$$ (9)

$$\text{MAPE}\%\;=\;\frac{\text{1}\text{0}\text{0}}{N}\;\sum_{t=\text{1}}^{N}\left|\frac{y_{i}-\hat{y_{i}}}{y_{i}}\right|$$ (10)

$$\text{sMAPE}\%=\;\frac{\text{1}\text{0}\text{0}}{N}\;\sum_{t=\text{1}}^{N}\frac{\text{2}\left|y_{i}-\hat{y_{i}}\right|}{\left|y_{i}\right|+\left|\hat{y_{i}}\right|}$$ (11)

$$\text{DA}\;\%=\;\frac{\text{1}\text{0}\text{0}}{N-\text{1}}\;\sum_{t=\text{2}}^{N}\text{1}\left[y_{i}-\;y_{i-\text{1}})(\hat{y_{i}}-\;{\hat{y}}_{i-\text{1}})\;\geq \text{0}\right]$$ (12)

$$WAPE\;(\%)=\text{1}\text{0}\text{0}\;\text{x}\;\;\;\;\;\frac{\sum_{t=\text{1}}^{N}\left|y_{i}-\hat{y_{i}}\right|}{\sum_{t=\text{1}}^{N}y_{i}}\;\;$$ (13)

$$Bias\;(Mean\;Error)=\frac{\text{1}}{N}\sum_{t=\text{1}}^{N}\left(\hat{y_{i}}{-y}_{i}\right)$$ (14)

Where:

${\text{y}}_{i}$ = actual/true value

$\hat{{\text{y}}_{\text{i}}}$ = predicted value

t   = time index,t = 1,…,N

N = number of paired observations/samples (test points)

$o_{t}$ = observed (actual)value at time t

$s_{t}$ = Predicted value at time t

1 [.] = indicator (1 if condition true, else 0)

### 3.7 Permutation feature importance

By randomly altering the order of each feature in the test set, the coefficient of determination (R2) is decreased to assess the influence of individual input features on model performance. In two distinct input configurations—one with thirteen variables, including seven additional technical indicators, and another with six core macroeconomic indicators—the feature importance is determined and evaluated using this model-agnostic approach. The study assessed how the relative value of features in predictive modeling is altered by the addition of technical indicators.

To predict absolute inaccuracy in the CCI, the GRU and LSTM models use Random Forest Regressors with an 80/20 train-test split. The Thirteen Features model adds technical indicators like RSI, EMA, and Bollinger Bands to the inputs, whereas the Six Features model solely examines macroeconomic and financial market data. Feature importance is determined by permutation analysis, and the results were exported and displayed similarly in both cases. The primary distinction is in scope: the Thirteen Features model incorporates additional technical elements to provide a more thorough evaluation than the Six Features model, which assesses the impact of macroeconomic variables.

### 3.8 Forecasting total civil works’ cost

The projected total cost of civil work is estimated by scaling the base cost using the ratio of forecasted to actual CCI. This relationship is mathematically expressed as equation (15):


$$\text{Fore\textasciitilde{}casted\textasciitilde{}Total\textasciitilde{}Cost}=\left(\frac{\text{Forecasted\textasciitilde{}CCI}}{\text{Baseline\textasciitilde{}CCI}}\right)\times \text{Initial\textasciitilde{}Estimated\textasciitilde{}Cost}$$ (15)

## 4 Results and analysis

### 4.1 Pearson correlation

**Fig 2:**
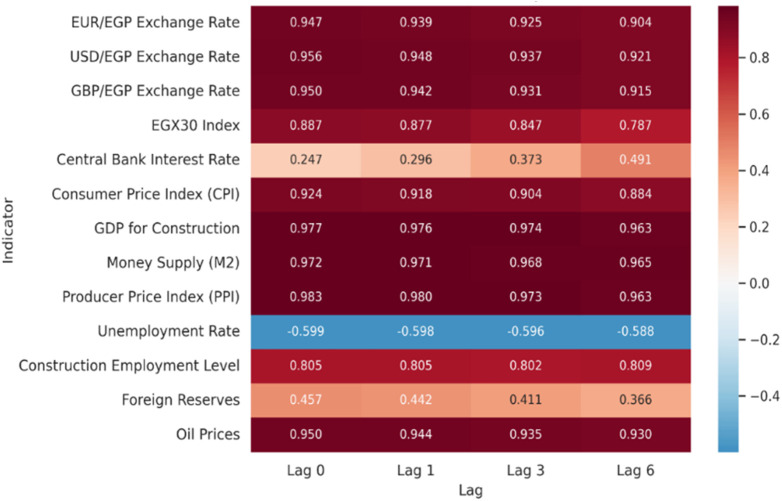
Pearson Correlation Matrix.

Using Pearson correlation coefficients, the relationship between the CCI and important Macroeconomic, Monetary, Foreign Exchange Market, equity market and Commodity/Energy market Indicators is investigated; unless otherwise indicated, corresponding p-values confirm statistical significance at the 0.01 level. A heat map that graphically depicts the direction and strength of these correlations over a number of time lags—0, 1, 3, and 6 months—is shown in (Fig 2) The color gradient allows for a clear visual comparison of the relative importance and temporal consistency of each variable, with deep blue denoting strong negative correlations and deep red denoting strong positive correlations. This graphic depiction successfully draws attention to both prevailing influences and new trends over time.

### 4.2 Spearman’s rank correlation

**Fig 3:**
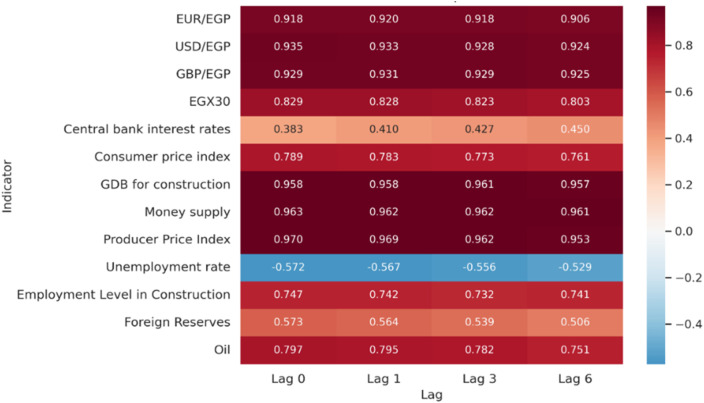
Spearman’s Rank Correlation.

A summary of the direction and strength of the relationships between the CCI and indicators from the macroeconomic, monetary, foreign exchange, equity, and commodity/energy markets using the Spearman rank correlation method. (Fig 3) illustrates this ranking, which highlights monotonic relationships by strength using the average of correlations across the four lags (0, 1, 3, and 6 months) (all statistically significant at p < 0.01 unless otherwise indicated).

### 4.3 Heat maps

**Fig 4:**
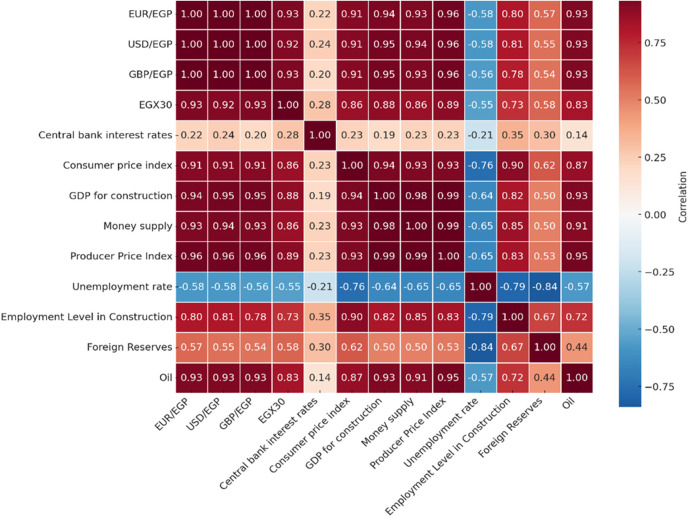
Heat Map Correlation.

The correlation analysis provides insights into Egypt’s the macroeconomic, monetary, foreign exchange, equity, and commodity/energy markets dynamics by highlighting important interrelationships among 13 indicators, as seen in (Fig 4). The heatmap shows a strong positive correlation between oil prices and exchange rates (EUR/EGP, USD/EGP, and GBP/EGP), showing that the global energy market influences currency trends. The money supply, PPI, and CPI—all indicators of inflation—also show strong intercorrelation, which is indicative of underlying inflationary pressures. The construction industry’s influence on market performance is highlighted by the positive correlation between EGX30 and Foreign Exchange Rates levels. On the other hand, the unemployment rate shows significant negative correlations with both foreign reserves and Employment Level Construction. The money supply and unemployment level have a negative correlation, but central bank interest rates only show a moderate relationship with the other indicators showing little impact.

### 4.4 Checking for multicollinearity

#### 4.4.1 Variance inflation factor (VIF).

**Fig 5:**
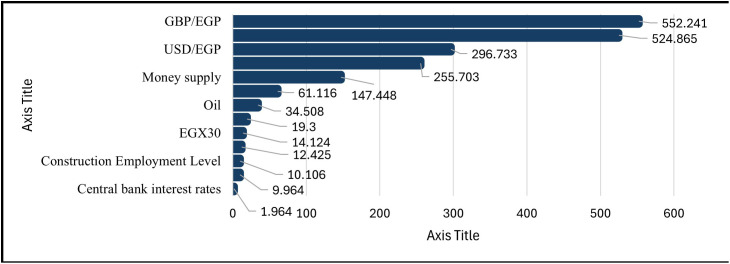
Variance Inflation Factor.

High VIF scores for several independent variables of macroeconomic and financial market indicators show significant multicollinearity, according to the Variance Inflation Factor (VIF) analysis summarized in (Fig 5). This shows that contributors have a strong linear relationship that can include model stability and regression coefficient reliability. The results prove how important it is to use variable transformation or dimensionality reduction techniques to increase the model’s interpretability.

#### 4.4.2 Significant inter-variable associations.

**Fig 6:**
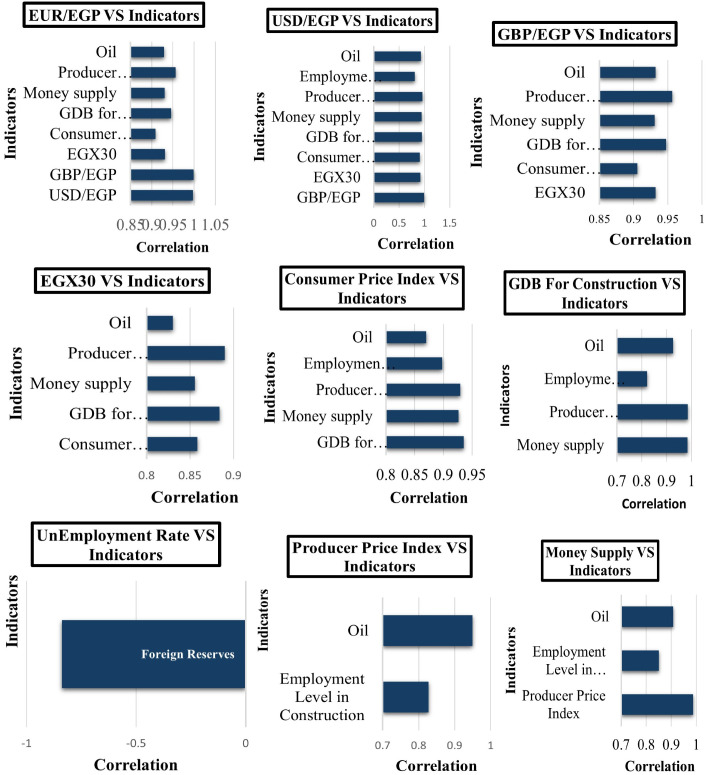
Correlation between several financial markets and macroeconomic data points (Indicators).

The cluster bar graphs in (Fig 6) illustrate the degree of correlation between several financial markets and macroeconomic data points. These graphs illustrate the relationships between important variables like inflation metrics, currency exchange rates, the performance of the construction industry, monetary aggregates, and international commodities like oil.

Strong co-movements are shown by high correlation values, which provide insightful information for forecasting and policy analysis. By clearly showing clusters of closely related variables, this visual approach improves comprehension and shows the foundation for more in-depth statistical analysis and model development.

### 4.5 Stepwise regression

Following the multicollinearity assessment, forward stepwise regression is applied to identify the most influential predictors of the CCI. This method refines the model by systematically including variables that significantly contribute to explaining CCI while excluding redundant or highly collinear ones. Table 2 presents the step-by-step inclusion of predictors and their corresponding impact on the model’s statistical performance.

**Table 2:** Result of Stepwise Regression.

Y(CCI)- with indicators	X1	X2	X3	X4	X5	X6	Adj. R-squared	Akaike Information Criterion (AIC)	Bayesian Information Criterion (BIC)
Producer Price Index	•						0.966	1685.228	1691.834
Producer Price Index + Foreign Reserves	•	•					0.972	1645.104	1655.014
Producer Price Index + Foreign Reserves + Consumer Price Index	•	•	•				0.976	1611.770	1624.983
Producer Price Index + Foreign Reserves + Consumer price index + EGX30	•	•	•	•			0.978	1595.317	1611.833
Producer Price Index + Foreign Reserves + Consumer price index + EGX30 + Oil	•	•	•	•	•		0.980	1581.967	1601.787
Producer Price Index + Foreign Reserves + Consumer price index + EGX30 + Oil + Money supply	•	•	•	•	•	•	0.981	1572.138	1595.261

### 4.6 Model development and evaluation

#### 4.5.1 GRU model.

**Fig 7:**
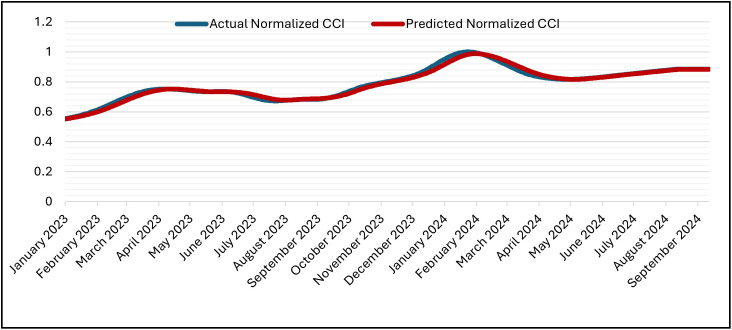
Actual vs. Predicted – Normalized CCI – Tested Data – GRU Model.

**Fig 8:**
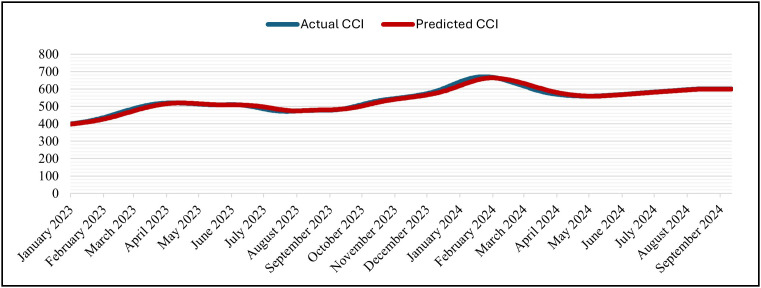
Actual vs. Predicted – Denormalized CCI – Tested Data – GRU Model.

**Fig 9:**
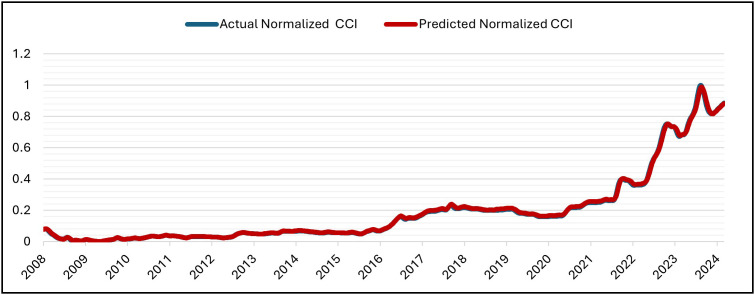
Actual vs. Predicted – Normalized CCI – Whole Data – GRU Model.

**Fig 10:**
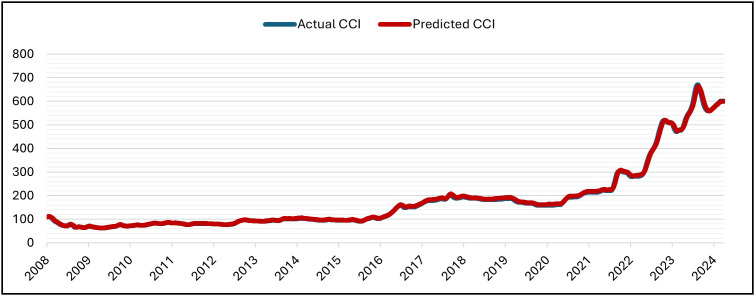
Actual vs. Predicted – Denormalized CCI – Whole Data – GRU Model.

The GRU was set up for 50 epochs, but it stopped early (patience = 5) at epoch 16 and recovered the best weights from epoch 11. With training MSE = 7.1141 × 10 ⁻ ⁶ at the same epoch, validation MSE dropped from 7.6139 × 10 ⁻ ⁴ (epoch 1) to at least 6.6533 × 10 ⁻ ⁵ (epoch 11; best epoch). Subsequent validation losses were epoch (12,13,14,15,16), all of which were higher than the epoch-11 minimum. As a result, early stopping and weight restoration to epoch 11 were triggered by the patience window, which covered epochs 12–16.. Over 16 epochs, the training time was approximately 2,030 s (≈ 33 min 50 s), with an average of ~126.9 s/epoch (range ~122–139 s). The hold-out split’s testing (inference) time was about 5 seconds (19 steps at about 0.247 seconds per step). In general, the GRU model is right and efficient at making predictions. This is shown by the close match between normalized predicted and actual CCI values during the testing period at the Tested Data (Fig 7) and its denormalized (Fig 8). It is also shown by the close match between normalized predicted and actual CCI values during the whole dataset (trained and tested periods) (Fig 9) and its denormalization (Fig 10).

#### 4.5.2 LSTM model.

**Fig 11:**
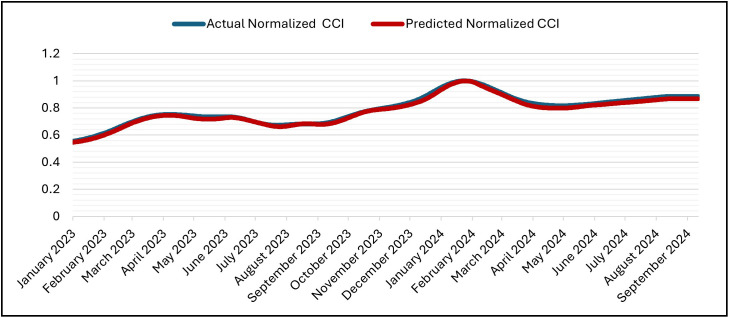
Actual vs. Predicted – Normalized CCI – Tested – LSTM Model.

**Fig 12:**
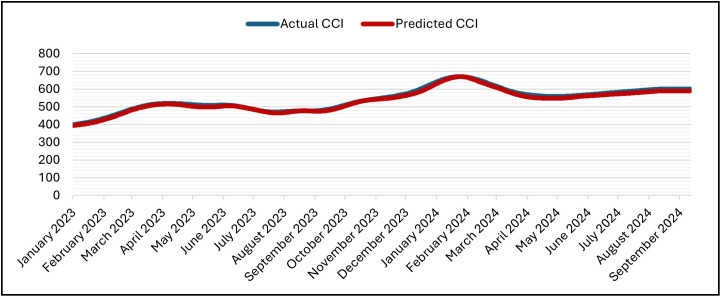
Actual vs. Predicted – Denormalized CCI – Tested – LSTM Model.

**Fig 13:**
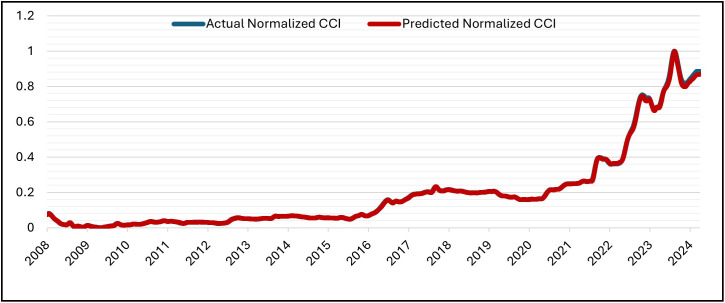
Actual vs. Predicted – Normalized CCI – Whole Data – LSTM Model.

**Fig 14:**
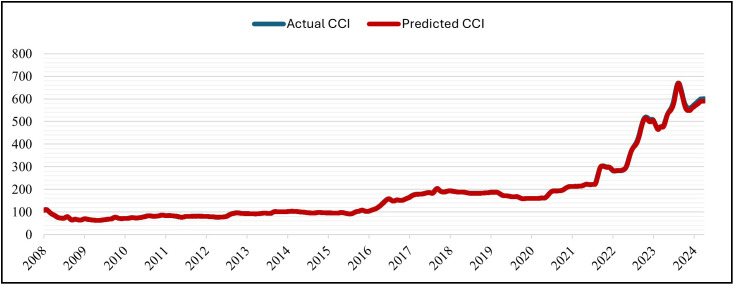
Actual vs. Predicted – Denormalized CCI – Whole Data – LSTM Model.

The LSTM was set up for 50 epochs with early stopping and a patience of 5. Validation loss went down from 1.2000 × 10 ⁻ ³ at epoch 1 to 4.2889 × 10 ⁻ ⁴ (epoch 2), 1.5497 × 10 ⁻ ⁴ (epoch 10), and then improved in steps at epochs (12,14,16,18, 22) and reached its minimum at epoch 25 with Validation Losses = 1.2687 × 10 ⁻ ⁵ (training MSE = 1.5663 × 10 ⁻ ⁵). After that, there was no more improvement, so training stopped at epoch 30 and the weights were restored to those from epoch 25 according to the patience window. The 30 epochs that were run took about 3,892 seconds (about 64 minutes and 52 seconds) in total, with an average of about 129.7 seconds per epoch (between 125 and 142 seconds). The inference on the hold-out split took about 5 seconds (19 steps at about 0.238 seconds per step). The predicted and actual CCI series stay near to each other during the test period (Fig 11) and its denormalization (Fig 12) and over the whole timeline (Fig 13) and its denormalization (Fig 14). This shows that LSTM model has great accuracy and is efficient in predicting.

### 4.7 Evaluation metrics

Key metrics are used to compare the predictive performance of Long Short-Term Memory (LSTM) and Gated Recurrent Unit (GRU) models. Table 3 below gives a summary of the results.

**Table 3:** Evaluation Metrics for LSTM Model and GRU Model.

Metric	LSTM Model	GRU Model	Goal	Margin	Margin %	Better Model	Note
MAE	0.01157	0.01034	Lower	0.00124	11.966%	GRU	
RMSE	0.01296	0.01386	Lower	−0.00090	−6.508%	LSTM	
MAPE%	1.46945	1.29972	Lower	0.16973	13.059%	GRU	
sMAPE %	1.48285	1.30455	Lower	0.17830	13.667%	GRU	
Directional Accuracy %	94.74576	93.72881	Higher	1.01695	1.085%	LSTM	
R^2^	0.98437	0.98212	Higher	0.00225	0.229%	LSTM	
Median Error	0.01197	0.00762	Closer to 0	0.00435	57.124%	GRU	
MSE	0.00017	0.00019	Lower	−0.00002	−12.591%	LSTM	
WAPE %	1.47406	1.31657	Minimize	0.15749	11.96%	GRU	
Bias (Mean Error)	−0.01153	−0.00303	≈ 0 (min |bias|)	0.00850	280.53%	GRU	

Using a hold-out test set (*N* = 591), both models achieve excellent accuracy; GRU Model achieves (r = 0.991; R² = 0.982; MAPE = 1.30%) while LSTM Model achieve (r = 0.998; R² = 00.984; MAPE = 1.47%). The GRU outperforms on 12/19 targeted criteria, led by superior calibration (α = 0.99947, β = 0.99614; both closest to 1), markedly lower typical errors (MAE = 0.01034; WAPE = 1.3166%; MAPE = 1.2997%; sMAPE = 1.3046%), and a much smaller bias magnitude (|bias| = 0.00303 vs 0.01153; ≈ 3.8 × smaller). In contrast, The LSTM is better when tracking signals and aligning variances are very important: it yields higher correlation (r = 0.9985), R² (0.9844), Directional Accuracy (94.75%), **γ** closer to 1, closer CV to the observed series, and slightly lower RMSE/MSE (RMSE = 0.01296; MSE = 0.00017), showing fewer big error spikes even though the average absolute errors are bigger.

### 4.8 Permutation feature importance

A permutation feature significance analysis is used to figure out how much each input variable contributes to the GRU and LSTM models make predictions. This method doesn’t depend on any one model. It randomly changes the values of individual features to see how they affect model performance, which is measured by changes in Mean Squared Error (MSE). This method makes it easy to find the most important predictors in the modeling framework clearly and easy to understand.

#### 4.7.1 GRU model.

**Fig 15:**
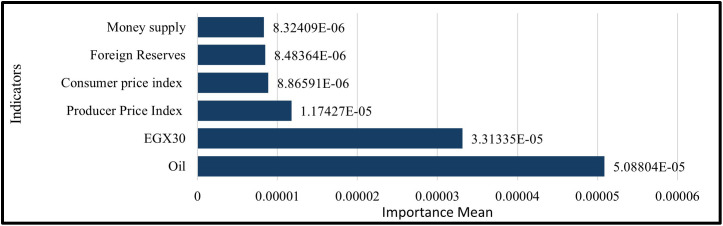
Permutation Feature Importance– Six Features– GRU Model.

With the Evaluation of Permutation feature importance (PFI) shows that the GRU Model relies most on oil prices (importance mean = 5.08804 × 10 ⁻ ⁵, ≈ 41.9% of total importance), followed by the EGX30 index (3.31335 × 10 ⁻ ⁵, ≈ 27.3%). The Producer Price Index (PPI) contributes 1.17427 × 10 ⁻ ⁵ (≈9.7%), then CPI 8.86591 × 10 ⁻ ⁶ (≈7.3%), foreign reserves 8.48364 × 10 ⁻ ⁶ (≈7.0%), and money supply 8.32409 × 10 ⁻ ⁶ (≈6.9%). Together, oil and EGX30 account for ≈69.2% of total importance, underscoring the model’s sensitivity to commodity and equity market conditions. Macroeconomic aggregates (PPI, CPI) and Monetary indicators (Foreign reserves, and money supply) have smaller yet small effects as shown in (Fig 15).

**Fig 16:**
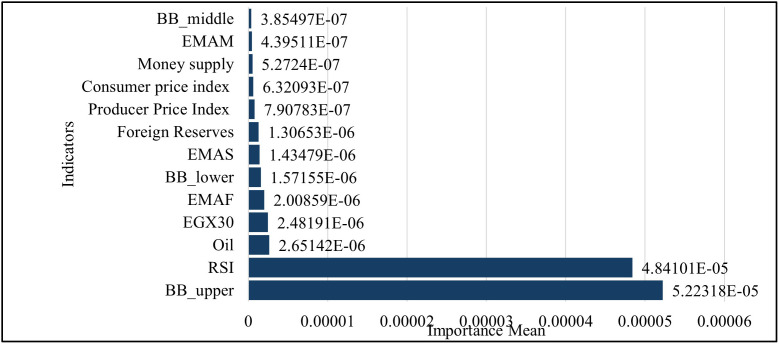
Permutation Feature Importance – Thirteen Features – GRU Model.

With Evaluation in adding the technical indicators in GRU Model to the six features to become thirteen features, the permutation feature importance (PFI) for the GRU indicates that technical indicators predominate model dependence. The Bollinger upper band (BB_upper) has the highest importance mean (5.22318 × 10 ⁻ ⁵; ≈ 45.5% of total), and the RSI (4.84101 × 10 ⁻ ⁵; ≈ 42.1%) comes in second. A second group of predictors adds a little: Oil (2.65142 × 10 ⁻ ⁶; ≈ 2.31%), EGX30 (2.48191 × 10 ⁻ ⁶; ≈ 2.16%), and the fast EMA (EMAF) (2.00859 × 10 ⁻ ⁶; ≈ 1.75%). The other features each add less than 1.5%: BB_lower (1.57155 × 10 ⁻ ⁶; ≈ 1.37%), EMAS (1.43479 × 10 ⁻ ⁶; ≈ 1.25%), Foreign Reserves (1.30653 × 10 ⁻ ⁶; ≈ 1.14%), PPI (7.90783 × 10 ⁻ ⁷; ≈ 0.69%), CPI (6.32093 × 10 ⁻ ⁷; ≈ 0.55%), Money Supply (5.27240 × 10 ⁻ ⁷; ≈ 0.46%), EMAM (4.39511 × 10 ⁻ ⁷; ≈ 0.38%), and BB_middle (3.85497 × 10 ⁻ ⁷; ≈ 0.34%). Overall, technical signals make up about 92.7% of the total importance, while macro-financial variables make up about 7.3%. This shows that short-horizon momentum and volatility cues (RSI, Bollinger bands, EMAs) are the main factors that affect the GRU’s predictions in this specification (Fig 16).

### 4.7.2 LSTM model

**Fig 17:**
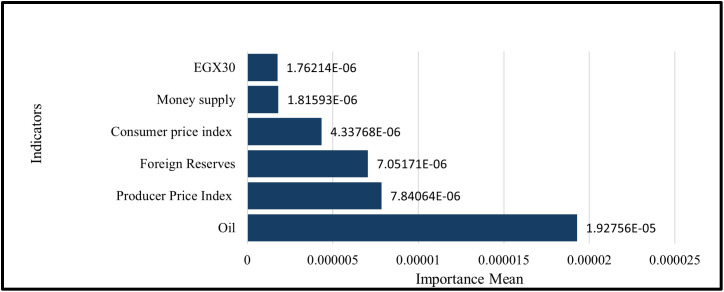
Permutation Feature Importance –Six Features – LSTM Model.

Permutation feature importance (PFI) for this LSTM Model shows that oil prices are the dominant driver (importance mean 1.92756 × 10 ⁻ ⁵, ≈ 45.8% of total). Next are the Producer Price Index (PPI) (7.84064 × 10 ⁻ ⁶, ≈ 18.6%) and foreign reserves (7.05171 × 10 ⁻ ⁶, ≈ 16.8%). CPI contributes moderately (4.33768 × 10 ⁻ ⁶, ≈ 10.3%), while money supply (1.81593 × 10 ⁻ ⁶, ≈ 4.3%) and EGX30 (1.76214 × 10 ⁻ ⁶, ≈ 4.2%) are comparatively small. Overall, the model relies mostly on commodity price changes (like oil) and price-level indicators (like the PPI and CPI). The equity market signal (EGX30) only plays a small role in this setup as shown in (Fig 17).

**Fig 18:**
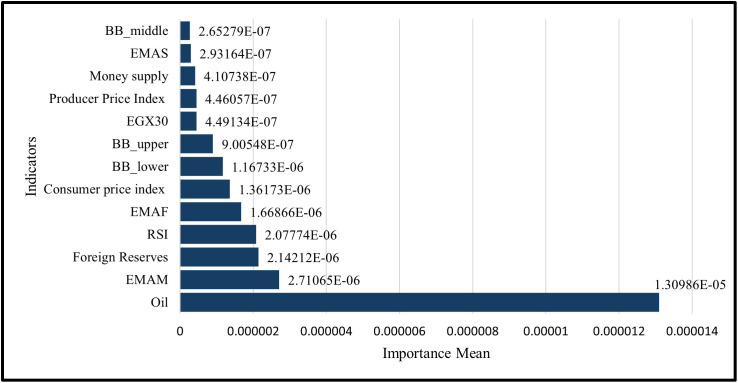
Permutation Feature Importance –Thirteen Features – LSTM Model.

With adding technical indicators in LSTM Model to the six features so it becomes thirteen features, the permutation feature importance (PFI) for this GRU setup shows a macro-led profile. Oil is the dominant driver (importance mean 1.30986 × 10 ⁻ ⁵, ≈ 48.5% of total). Next come the EMAM (2.71065 × 10 ⁻ ⁶, ≈ 10.0%), foreign reserves (2.14212 × 10 ⁻ ⁶, ≈ 7.9%), RSI (2.07774 × 10 ⁻ ⁶, ≈ 7.7%), and EMAF (1.66866 × 10 ⁻ ⁶, ≈ 6.2%). Moderate contributors are CPI (1.36173 × 10 ⁻ ⁶, ≈ 5.0%), Bollinger lower band (1.16733 × 10 ⁻ ⁶, ≈ 4.3%), and Bollinger upper band (9.00548 × 10 ⁻ ⁷, ≈ 3.3%). The remaining indicators each contribute ≲2%: EGX30 (4.49134 × 10 ⁻ ⁷, ≈ 1.7%), PPI (4.46057 × 10 ⁻ ⁷, ≈ 1.7%), money supply (4.10738 × 10 ⁻ ⁷, ≈ 1.5%), EMAS (2.93164 × 10 ⁻ ⁷, ≈ 1.1%), and Bollinger middle (2.65279 × 10 ⁻ ⁷, ≈ 1.0%). Overall, macro-financial variables account for ~66.3% of total importance (driven largely by oil), while technical signals contribute ~33.7%, showing that price-level and liquidity conditions dominate with technical momentum/volatility features providing secondary explication power as shown in (Fig 18).

#### 4.7.3 Comparison of the feature importance in the GRU model and LSTM model.

A permutation feature importance analysis is performed to assess the response of GRU and LSTM models to various macroeconomic and financial market inputs by evaluating the effect of random permutation of each feature on prediction error. This method showed how macroeconomic factors, like the money supply and foreign reserves, are more important than market-based factors, like oil prices and stock indices. Table 3 shows comparative results, which show how each model captures temporal dependencies and prioritize features. This is especially true after adding technical indicators, which gives us a better idea of how the models make decisions, In the Permutation of the importance of the Six Features, The GRU model better captures how the equity market works, while the LSTM model focuses on price-level and liquidity proxies as shown in Table 4.While In the Permutation of the importance of the Thirteen Features. The GRU mostly looks at short-term momentum and volatility regimes, like Bollinger bands and the RSI. The LSTM, on the other hand, looks more at trend and level information from macro-liquidity signals (EMAs, reserves) and commodities (Oil) as shown in Table 5.

**Table 4:** Comparison of Permutation Feature Importance between GRU and LSTM Models for Six Features.

Feature	GRU Model		LSTM Model	
	Importance Mean	Rank	Importance Mean	Rank
Oil	5.08804E-05	1	1.92756E-05	1
EGX30	3.31335E-05	2	1.76214E-06	6
Producer Price Index	1.17427E-05	3	7.84064E-06	2
Consumer price index	8.86591E-06	4	4.33768E-06	4
Foreign Reserves	8.48364E-06	5	7.05171E-06	3
Money supply	8.32409E-06	6	1.81593E-06	5

**Table 5:** Comparison of Permutation Feature Importance between GRU and LSTM Models for Thirteen Features.

Feature	GRU Model		LSTM Model	
	Importance Mean	Rank	Importance Mean	Rank
BB_upper	5.22318E-05	1	9.00548E-07	8
RSI	4.84101E-05	2	2.07774E-06	4
Oil	2.65142E-06	3	1.30986E-05	1
EGX30	2.48191E-06	4	4.49134E-07	9
EMAF	2.00859E-06	5	1.66866E-06	5
BB_lower	1.57155E-06	6	1.16733E-06	7
EMAS	1.43479E-06	7	2.93164E-07	12
Foreign Reserves	1.30653E-06	8	2.14212E-06	3
Producer Price Index	7.90783E-07	9	4.46057E-07	10
Consumer price index	6.32093E-07	10	1.36173E-06	6
Money supply	5.2724E-07	11	4.10738E-07	11
EMAM	4.39511E-07	12	2.71065E-06	2
BB_middle	3.85497E-07	13	2.65279E-07	13

### 4.9 Forecasting of the total civil works’ cost

The total cost of civil work for the construction project in question was 72,345,000 EGP as of February 2022.

**Fig 19:**
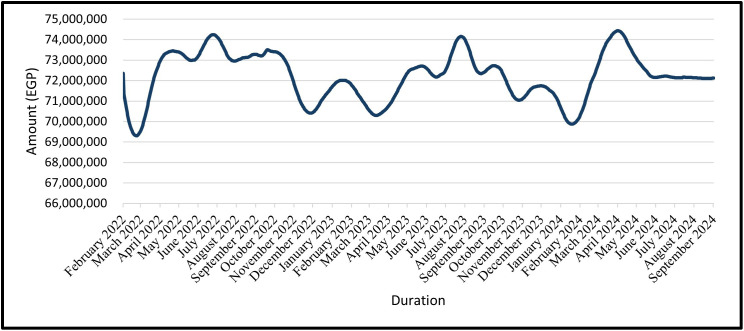
GRU Model – Forecasted Civil Work’s Cost for Entire Data (Trained Data & Tested Data).

**Fig 20:**
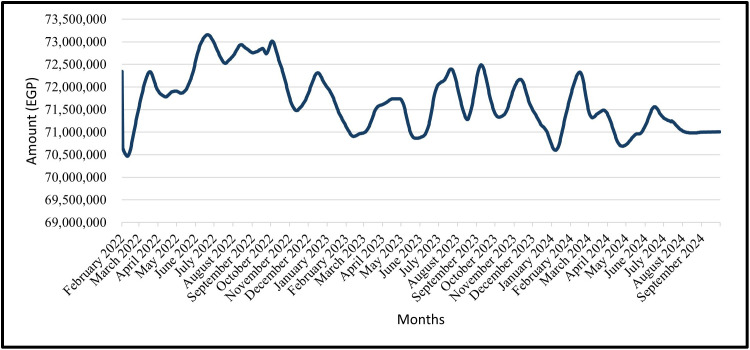
LSTM Model – Forecasted Civil Work’s Cost for Entire Data (Trained Data & Tested Data).

The predicted cost of civil works for the trained data is shown from February 2022 to December 2022 and the tested data from January 2023 to September 2024 for the GRU Model in (Fig 19) and the LSTM Model in (Fig 20).

**Fig 21:**
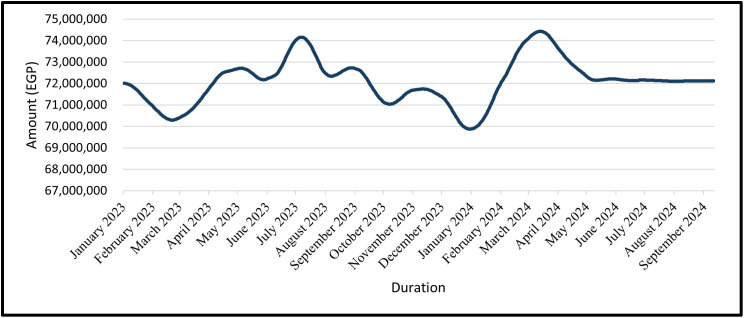
GRU Model – Forecasted Civil Work’s Cost for Tested Data.

**Fig 22:**
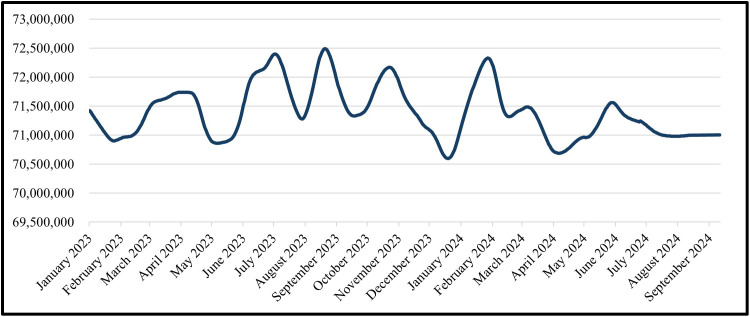
LSTM Model – Forecasted Civil Work’s Cost for Tested Data.

The predicted cost of civil work is shown for the time period of the tested data, which is from January 2023 to September 2024 (Fig 21) shows this for the GRU Model and (Fig 22) shows for the LSTM Model.

## 5 Conclusion, limitations and recommendations

### 5.1 Conclusion

Using a hold-out test set (N = 591), both deep-learning approaches deliver highly accurate CCI forecasts and cost projections. The GRU is the better all-round forecaster, winning 12/19 targeted criteria and excelling when the objective is to minimize typical error and bias: lower MAE (0.01034), WAPE (1.3166%), MAPE (1.2997%), sMAPE (1.3046%), and a much smaller |bias| (0.00303 vs 0.01153). In contrast, the LSTM is preferred when the goal is to track the signal and variance dynamics as tightly as possible: it attains higher correlation with the observations (r ≈ 0.9985), slightly higher R² (≈ 0.9844), better directional accuracy (94.75%), and marginally lower RMSE/MSE (0.01296/ 0.00017), indicating fewer large error spikes despite somewhat larger average absolute errors.

Permutation Feature Importance (PFI) explains why the models are different. Both models are sensitive to oil with six features, but GRU gives much more weight to EGX30 (equity conditions) and LSTM gives more weight to price-level and liquidity proxies (PPI, Reserves, CPI). Profiles diverge more when there are thirteen features: Bollinger’s upper band and RSI together make up about 92.7% of GRU’s total importance, which means that it relies on short-term momentum and volatility regimes. LSTM is still led by macro factors, with Oil in the lead, followed by EMAM, Reserves, and RSI. This is because macro-liquidity and commodity conditions affect trends and levels.

For civil-works cost forecasting, the baseline total was EGP 72,345,000 (Feb-2022). Across the training period (Feb-2022 → Dec-2022) and the testing period (Jan-2023 → Sep-2024), both models reproduce the seen path closely (Figs 17–20). In practice:

- Choose GRU, when making budgets, pricing change orders, or setting monthly accruals, because it has lower absolute percentage error and less bias, which helps to keep financial slippage to a minimum.
- Choose LSTM for early warning and turning point detection, where tracking variance and directional accuracy are more important for risk flags and management interventions.

The results show that Egypt’s construction costs, as measured by the CCI, are affected by both macro-price levels/liquidity and market-based signals. However, the relative importance of these factors changes depending on the model class and feature set. This duality is useful for governance: a macro-led LSTM view helps with planning and setting indexation policy for the medium term, while a technical-led GRU view helps with short-term execution and cash-flow control.

The proposed monthly CCI forecasting model offers actionable value across the project ecosystem. For public owners and ministries (e.g., Infrastructure development, Housing, Transport) and statistical authorities such as CAPMAS, it can inform escalation policies, support the publication of forward-looking cost indices, and provide an evidence base for the evaluation of Public–Private Partnership affordability. Private developers and project sponsors may improve budgeting accuracy, stage progress payments more effectively, size contingencies, and time procurement to predicted cost cycles. Contractors and subcontractors can get materials and hedging positions before price tenders and expected uptrends by using risk allowances that are based on numbers. Quantity surveyors and cost consultants can use model-based evidence to write index-linked contract clauses, benchmark variations, and back up change orders.

Finally, even though performance is good, it’s a good idea to re-estimate regularly to protect against regime shifts (policy changes, commodity shocks, FX realignments) and data revisions. Deployment should be accompanied by tests.

### 5.2 Limitations

Although providing great positive results, this study has several limitations. The model’s effectiveness only works in Egypt’s unique construction industry, so it might not be useful in places with different construction rules or economic and financial market structures. International economic shocks were not explicitly considered, thereby constraining the broader applicability of the conclusions.

The current design puts more emphasis on how accurate predictions are than on showing how things happen. High multicollinearity and shared macro shocks can make correlations look bigger and make models fit in the same way. Using contemporaneous features makes it harder to figure out the direction of the relationship; lag structure hasn’t been formally modeled yet. This study does not include structural identification, which means figuring out the difference between direct and mediated effects

### 5.3 Recommendations for future research

This research provides a solid foundation for employing intelligent forecasting models, particularly GRU and LSTM networks, to estimate construction expenses. To enhance the model’s applicability across diverse contexts, later research should examine its functionality in various geographic and economic environments. With more effort, these models could enhance stakeholder engagement and transform cost management by increasing flexibility in response to global market fluctuations.

Moreover, More Extensions in GRU Model & LSTM Model by adding more indicators such as interest rates across different maturities (government bond yields), equity market indices, credit spreads, or capital flows and test them in prediction the CCI.

Additionally, the examination of direct and indirect pathways will enhance the framework through Path Analysis/Structural Equation Modeling, enabling the (i) specification of theoretically grounded pathways among observed variables, (ii) quantification of mediated effects, and (iii) consideration of measurement error. This method is better for figuring out if CCI is mostly an outcome variable that reacts to macro-financial conditions or if it can be modeled as part of a feedback system.

## Supporting information

S1 Data Minimal Data set. (DOCX)
