# Supplementary material for: Macroeconomic-aware forecasting of construction costs in developing countries: Using gated recurrent unit and long short-term memory deep learning framework
Source: PLoS One. 2025 Oct 9;20(10):e0333189. doi: 10.1371/journal.pone.0333189 (PMC12510611; doi:10.1371/journal.pone.0333189)
Supplement: S1 Data — (DOCX) [file pone.0333189.s001.docx]

Comparison of Permutation Feature Importance between GRU and LSTM Models for Six Features

| Feature | GRU Model | | LSTM Model | |
| --- | --- | --- | --- | --- |
|  | Importance Mean | Rank | Importance Mean | Rank |
| Oil | 5.08804E-05 | 1 | 1.92756E-05 | 1 |
| EGX30 | 3.31335E-05 | 2 | 1.76214E-06 | 6 |
| Producer Price Index | 1.17427E-05 | 3 | 7.84064E-06 | 2 |
| Consumer price index | 8.86591E-06 | 4 | 4.33768E-06 | 4 |
| Foreign Reserves | 8.48364E-06 | 5 | 7.05171E-06 | 3 |
| Money supply | 8.32409E-06 | 6 | 1.81593E-06 | 5 |

Result of Stepwise Regression

| **Y(CCI)- with indicators** | **X1** | **X2** | **X3** | **X4** | **X5** | **X6** | **Adj. R-squared** | **Akaike Information Criterion (AIC)** | **Bayesian Information Criterion (BIC)** |
| --- | --- | --- | --- | --- | --- | --- | --- | --- | --- |
| Producer Price Index | • |  |  |  |  |  | 0.966 | 1685.228 | 1691.834 |
| Producer Price Index + Foreign Reserves | • | • |  |  |  |  | 0.972 | 1645.104 | 1655.014 |
| Producer Price Index + Foreign Reserves + Consumer Price Index | • | • | • |  |  |  | 0.976 | 1611.770 | 1624.983 |
| Producer Price Index + Foreign Reserves + Consumer price index + EGX30 | • | • | • | • |  |  | 0.978 | 1595.317 | 1611.833 |
| Producer Price Index + Foreign Reserves + Consumer price index + EGX30 + Oil | • | • | • | • | • |  | 0.980 | 1581.967 | 1601.787 |
| Producer Price Index + Foreign Reserves + Consumer price index + EGX30 + Oil + Money supply | • | • | • | • | • | • | 0.981 | 1572.138 | 1595.261 |

Evaluation Metrics for LSTM Model and GRU Model

| Metric | LSTM Model | GRU Model | Goal | Margin | Margin % | Better Model | Note |
| --- | --- | --- | --- | --- | --- | --- | --- |
| MAE | 0.01157 | 0.01034 | Lower | 0.00124 | 11.966% | GRU |  |
| RMSE | 0.01296 | 0.01386 | Lower | -0.00090 | -6.508% | LSTM |  |
| MAPE% | 1.46945 | 1.29972 | Lower | 0.16973 | 13.059% | GRU |  |
| sMAPE % | 1.48285 | 1.30455 | Lower | 0.17830 | 13.667% | GRU |  |
| Directional Accuracy % | 94.74576 | 93.72881 | Higher | 1.01695 | 1.085% | LSTM |  |
| R^2^ | 0.98437 | 0.98212 | Higher | 0.00225 | 0.229% | LSTM |  |
| Median Error | 0.01197 | 0.00762 | Closer to 0 | 0.00435 | 57.124% | GRU |  |
| MSE | 0.00017 | 0.00019 | Lower | -0.00002 | -12.591% | LSTM |  |
| WAPE % | 1.47406 | 1.31657 | Minimize | 0.15749 | 11.96% | GRU |  |
| Bias (Mean Error) | -0.01153 | -0.00303 | ≈ 0 (min \|bias\|) | 0.00850 | 280.53% | GRU |  |

Comparison of Permutation Feature Importance between GRU and LSTM Models for Thirteen Features.

| Feature | GRU Model | | LSTM Model | |
| --- | --- | --- | --- | --- |
|  | Importance Mean | Rank | Importance Mean | Rank |
| BB_upper | 5.22318E-05 | 1 | 9.00548E-07 | 8 |
| RSI | 4.84101E-05 | 2 | 2.07774E-06 | 4 |
| Oil | 2.65142E-06 | 3 | 1.30986E-05 | 1 |
| EGX30 | 2.48191E-06 | 4 | 4.49134E-07 | 9 |
| EMAF | 2.00859E-06 | 5 | 1.66866E-06 | 5 |
| BB_lower | 1.57155E-06 | 6 | 1.16733E-06 | 7 |
| EMAS | 1.43479E-06 | 7 | 2.93164E-07 | 12 |
| Foreign Reserves | 1.30653E-06 | 8 | 2.14212E-06 | 3 |
| Producer Price Index | 7.90783E-07 | 9 | 4.46057E-07 | 10 |
| Consumer price index | 6.32093E-07 | 10 | 1.36173E-06 | 6 |
| Money supply | 5.2724E-07 | 11 | 4.10738E-07 | 11 |
| EMAM | 4.39511E-07 | 12 | 2.71065E-06 | 2 |
| BB_middle | 3.85497E-07 | 13 | 2.65279E-07 | 13 |
